# Multidisciplinary Airway Management and Postoperative Planning in a Pediatric Patient With Unrepaired Treacher-Collins Syndrome: A Case Report

**DOI:** 10.7759/cureus.87655

**Published:** 2025-07-10

**Authors:** Lauren E Rein, Alexis McQuitty, Thong Nguyen

**Affiliations:** 1 Anesthesiology, University of Texas Medical Branch at Galveston, Galveston, USA

**Keywords:** cleft lip and palate surgery, combination of glidescope and fiberoptic intubation, mandibular hypoplasia, ovassapian oral airway device, treacher-collins syndrome

## Abstract

Treacher-Collins syndrome (TCS) presents significant challenges in airway management due to craniofacial abnormalities that often worsen with age. We describe the anesthetic management of a three-year-old male with TCS and no history of corrective surgery. Ketamine was used to facilitate vascular access and achieve adequate anesthetic depth. After three unsuccessful intubation attempts, an adult-sized Ovassapian airway was employed to guide a fiberoptic bronchoscope beneath the epiglottis and through the vocal cords, resulting in successful intubation. Postoperative airway management was optimized through multidisciplinary collaboration, highlighting the importance of preoperative planning and team-based decision-making in complex pediatric airway cases.

## Introduction

Treacher-Collins syndrome (TCS) is a rare congenital disorder of craniofacial development caused by mutations in the *TCOF1* gene, with an estimated incidence of approximately one in 50,000 live births [1]. Individuals with TCS often undergo multiple reconstructive surgeries, which are rarely fully corrective [1]. Characteristic craniofacial abnormalities, such as zygomatic hypoplasia, micrognathia, and possible cleft palate/lips, pose significant challenges for airway management, particularly for anesthesiologists. The severity of these anomalies varies widely among patients, necessitating individualized anesthetic and airway strategies.

There are still limited reports detailing effective airway management techniques in pediatric patients with TCS. Here, we present a case of a three-year-old male with unrepaired TCS, highlighting a multidisciplinary and fiberoptic-based approach to successful airway management.

## Case presentation

The patient is a three-year-old boy from Peru with a diagnosis of TCS. He communicated exclusively through sign language. Notably, he required chin elevation while eating or drinking to prevent leakage, suggestive of feeding difficulties and impaired growth. A prior attempt at cleft lip and palate repair was aborted due to intraoperative bronchospasm and the unavailability of a pediatric intensive care unit (PICU) at the time. Given these challenges, he presented to our facility for surgical repair of his cleft lip and palate.

**Figure 1:**
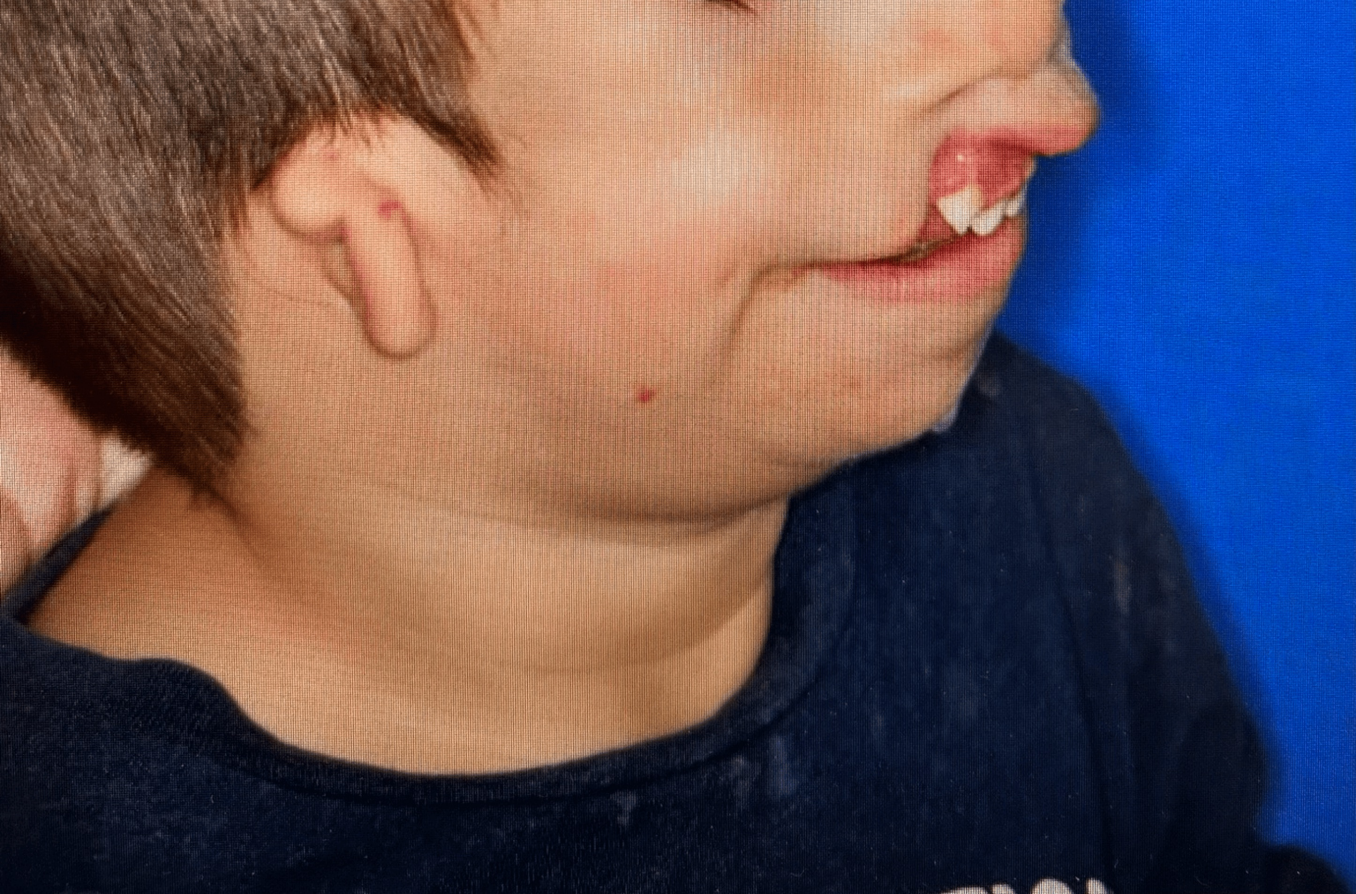
Picture of the patient in sagittal view showing microtia, micrognathia, mandibular hypoplasia, and cleft lip

**Figure 2:**
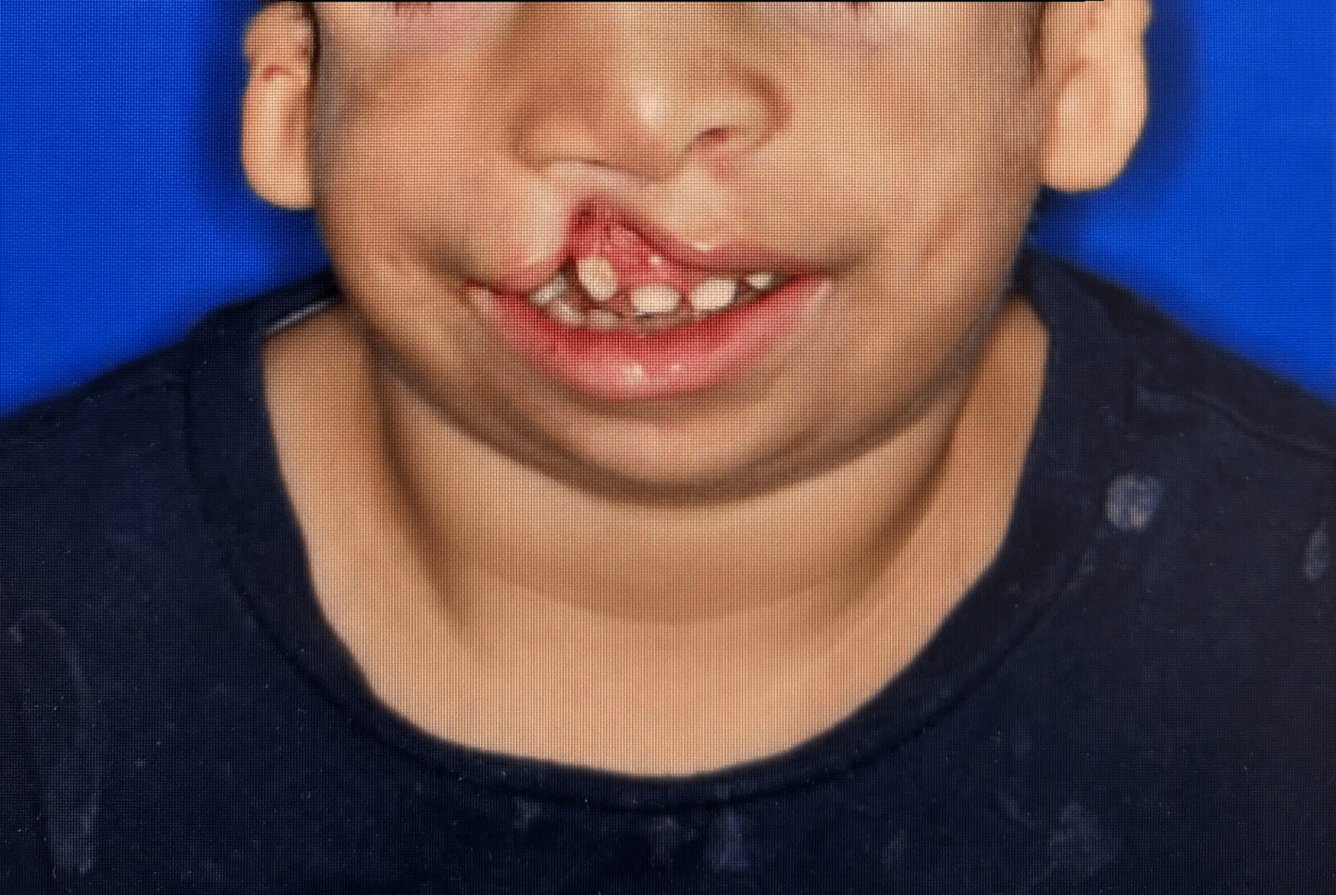
Picture of the patient in coronal view showing Treacher-Collins syndrome features

The patient weighed 13 kg and was non-verbal. He demonstrated characteristic features of TCS, including microtia, midface hypoplasia, micrognathia, mandibular hypoplasia, and a cleft lip and palate. Figures 1, 2 showed these features. 

Preoperative intramuscular ketamine injection was administered to facilitate separation from his parents and allow for vascular access. He subsequently received 0.08 mg of glycopyrrolate and incremental intravenous doses of ketamine. Once adequate anesthetic depth was achieved, intubation was attempted using a Glidescope with a hyperangulated blade. Despite optimal positioning and external laryngeal manipulation, visualization remained limited with a Cormack-Lehane grade IV view. 

A nasal fiberoptic approach was then attempted; however, anatomical landmarks were difficult to identify, and the epiglottis and vocal cords could not be visualized. A tongue stitch was placed by the surgeon to improve tongue retraction, and an additional provider provided jaw thrust, yet visualization remained inadequate. A low-lying, omega-shaped epiglottis closely approximated the vocal cords, obscuring the glottic opening. Ultimately, with the use of an adult-sized Ovassapian airway, the fiberoptic bronchoscope was advanced beneath the epiglottis and through the vocal cords, achieving successful intubation. Despite multiple attempts that lasted about 45 minutes in total, the patient still maintained an oxygen saturation of 95-98% and his vital signs were stable. 

Anesthesia was maintained with sevoflurane, along with remifentanil and dexmedetomidine infusions for analgesia and to promote a smooth emergence. Dexamethasone (0.5 mg/kg) and ondansetron (0.15 mg/kg) were administered to reduce the risk of postoperative nausea, vomiting, and airway swelling. The patient remained hemodynamically stable throughout the five-hour procedure, which was completed without complications. The estimated blood loss was 20 mL.

Postoperatively, a multidisciplinary discussion was held regarding airway management. It was decided to attempt an awake extubation in the operating room, with an emergency airway cart (including a cricothyrotomy kit) and a pediatric ENT available at the bedside. Once the patient was awake and vigorous, extubation was successfully performed. The tongue stitch remained in place to support airway patency. He was transitioned to high-flow nasal cannula, positioned in a sniffing posture, and transferred to the PICU for close monitoring.

## Discussion

TCS presents significant challenges for anesthesia induction and airway management, particularly in pediatric patients. Craniofacial abnormalities in TCS, including a smaller cranial base, maxilla, and nose, contribute to upper airway narrowing, with the most pronounced morphological changes occurring between ages seven and 18 [2]. As patients grow, mandibular hypoplasia and maxillomandibular dysmorphology typically worsen, further complicating airway management [3]. In their study, Barrero et al.(2024) showed that higher mandibular retrusion and ramal hypoplasia scores were strongly correlated with difficult airway (p < 0.001), and a majority of patients with higher scores (grade III or IV) were tracheostomy-dependent at some point [4]. Hence, early evaluation of the mandible and ramal-condyle complex is crucial in TCS.

While no universally validated treatment timeline exists, tracheostomy or mandibular distraction osteogenesis (MDO) is typically reserved for severe cases and performed shortly after birth [5]. Craniofacial surgeons anecdotally prefer initial distraction surgery between the ages of eight and 10, followed by definitive orthognathic surgery in late adolescence, when feasible. This preference stems from the high relapse rates and need for repeated distractions associated with early MDO in TCS patients [5]. Ali-Khan et al.(2018)* *found that the success rate of tracheostomy avoidance or decannulation within one year after first MDO is significantly lower in TCS than Pierre Robin sequence patients (21% versus 62%) [6]. The repeat rate of MDO in TCS patients was 46% as compared to 8% [6]. For our patient, MDO was not the option due to condylar aplasia. 

Due to the patient's lack of prior surgical interventions and difficult airway features, the goal was to maintain spontaneous ventilation. Ketamine is the induction agent of choice to achieve an adequate depth of anesthesia. Despite a 45-minute intubation attempt using multiple approaches and providers, the patient continued spontaneous breathing with 100% oxygen saturation. Ketamine, a dissociative anesthetic, provides both sedation and analgesia. Unlike other induction agents, it maintains normal pharyngeal-laryngeal reflexes and enhances skeletal muscle tone, helping to preserve airway patency [7]. These properties make ketamine an optimal choice for induction in pediatric patients with known difficult airways. Iravani and Wald (2008) demonstrated the use of ketamine and dexmedetomidine as induction agents in a pediatric TCS patient [8]. While this combination is a viable option, we elected not to use it due to the risk of oversedation. Due to the patient's lack of prior surgical interventions and difficult airway features, the goal was to maintain spontaneous ventilation. Ketamine is the induction agent of choice to achieve an adequate depth of anesthesia. Despite a 45-minute intubation attempt using multiple approaches and providers, the patient continued spontaneous breathing with 100% oxygen saturation. Ketamine, a dissociative anesthetic, provides both sedation and analgesia. Unlike other induction agents, it maintains normal pharyngeal-laryngeal reflexes and enhances skeletal muscle tone, helping to preserve airway patency [7]. These properties make ketamine an optimal choice for induction in pediatric patients with known difficult airways. Iravani and Wald (2008) demonstrated the use of ketamine and dexmedetomidine as induction agents in a pediatric TCS patient [8]. While this combination is a viable option, we elected not to use it due to the risk of oversedation.

Currently, there is a lack of comprehensive comparative studies evaluating the success rates of various intubation techniques in patients with TCS. A retrospective study by Hosking et al. (2012) found that 41% of intubations required alternative techniques beyond conventional direct laryngoscopy in TCS patients. Among these, the laryngeal mask airway (LMA) proved to be particularly useful, alongside other techniques such as video laryngoscopy and fiberoptic intubation, performed orally, nasally, or through an LMA [9]. Only a few case reports demonstrated the use of the tongue stitch. This case introduced the novel use of tongue stitch in combination with an Ovassapian device to facilitate fiberoptic intubation. Table 1 summarizes the different approaches used in our case.

**Table 1:** Description and outcome of different approaches for intubation in this case

Attempt	Method of intubation	Cormack-Lehane grade	Result
1	Glidescope with a hyperangulated blade	IV	Failed
2	Nasal fiberoptic approach	IV	Failed
3	Tongue stitch + jaw thrust + oral fiberoptic	IV	Failed
4	Tongue stitch + Ovassapian airway + oral fiberoptic	III	Successful

The extreme difficulty of intubation and the risk of postoperative respiratory failure raised a concern for the need for an intraoperative tracheostomy. After a multidisciplinary discussion was held, the consensus was to proceed with controlled, awake extubation, accompanied by a tongue-lip adhesion procedure to mitigate the risk of tongue-based airway obstruction. An ENT surgeon was present for emergent airway intervention if needed. The reason to delay tracheostomy is that it would result in prolonged hospital stay and increased long-term care needs, posing challenges for the patient's family, who is from another country. The postoperative plan includes close monitoring and proceeding with a polysomnography to assess the severity of obstructed sleep apnea (OSA). 

This approach aligns with the findings from Rizzi et al.* *(2017), who noted that tracheostomy is typically reserved for severe OSA (apnea-hypopnea index [AHI] >10) with persistent airway obstruction unresponsive to less invasive interventions [10]. Their study also emphasized that the majority of children requiring tracheostomy had underlying craniofacial abnormalities, reinforcing the tailored decision-making in syndromic patients like ours. 

## Conclusions

TCS poses challenges in pediatric anesthesia and craniofacial surgery. This report describes our management of a particularly complex case involving a child with TCS who had not undergone any previous surgical corrections. Our successful approach in addressing airway difficulties was founded on two pivotal strategies. First, the exclusive use of ketamine as the induction agent ensured that spontaneous ventilation was maintained. Second, the use of tongue suturing in conjunction with an Ovassapian oral airway device facilitated effective fiberoptic intubation. Finally, the multidisciplinary discussion was crucial in decision-making for postoperative airway management.
